# Persistent Subretinal Fluid in a Case With Central Serous Chorioretinopathy and Progressive Retinal Changes: An 18‐Month Longitudinal Case Report

**DOI:** 10.1155/crop/7754197

**Published:** 2026-06-30

**Authors:** Ryoh Funatsu, Naohisa Mihara, Hiroto Terasaki

**Affiliations:** ^1^ Department of Ophthalmology, Graduate School of Medical and Dental Sciences, Kagoshima University, Kagoshima, Japan, kagoshima-u.ac.jp

**Keywords:** central serous chorioretinopathy, optical coherence tomography, retina

## Abstract

**Introduction:**

This study is aimed at reporting detailed longitudinal changes in best‐corrected visual acuity (BCVA) and retinal structure in a patient with central serous chorioretinopathy (CSC) and persistent serous retinal detachment over one and a half years.

**Case Report:**

A man in his 40s presented with unilateral mild vision loss. On the initial examination, the BCVA was 20/25 OS. Optical coherence tomography (OCT) revealed macular serous retinal detachment, and fluorescein angiography identified two focal leakage spots within this area, leading to the diagnosis of CSC. Despite the persistence of serous retinal detachment after several focal photocoagulations, the patient refused photodynamic therapy, which resulted in the serous retinal detachment remaining for 18 months. In the affected eye, the BCVA and outer nuclear layer (ONL) thickness were initially 20/25 and 86 *μ*m, respectively. These values subsequently deteriorated to 20/67 and 73 *μ*m at 6 months and further decreased to 20/200 and 46 *μ*m at 18 months. The reflectivity of the photoreceptor layer progressively increased, leading to the formation of intraretinal hyperreflective foci 18 months from baseline.

**Conclusions:**

A patient with CSC and persistent serous retinal detachment may present a relatively rapid decline in BCVA and progressive retinal damage. The chronicity of CSC is associated with specific structural findings, such as thinning of the ONL and photoreceptor layer and increased photoreceptor reflectivity leading to intraretinal hyperreflective foci.

## 1. Introduction

Central serous chorioretinopathy (CSC) is common in middle‐aged people and is characterized by serous retinal detachment in the macula [1]. In CSC, visual acuity often remains preserved in the early stages, and the rate of visual decline is typically gradual [2, 3]. Nevertheless, approximately 13% of patients with chronic CSC develop legal blindness in the long term [2].

The recurrence of serous retinal detachment and poor visual acuity in CSC are associated with irreversible structural damage to the retina, such as outer nuclear layer (ONL) thinning, photoreceptor loss, cystoid macular degeneration, and retinal pigment epithelial atrophy [4–6]. However, the evolution of these longitudinal structural changes during persistent serous retinal detachment has not been fully characterized.

In this report, we present detailed longitudinal changes in retinal structure and visual acuity in a patient with chronic CSC and long‐term persistent serous retinal detachment. This case provided a unique opportunity to observe the effects of prolonged serous retinal detachment exposure on visual function and the retina, which cannot be assessed in an interventional study, as the patient declined treatment.

## 2. Case Presentation

**Figure 1:**
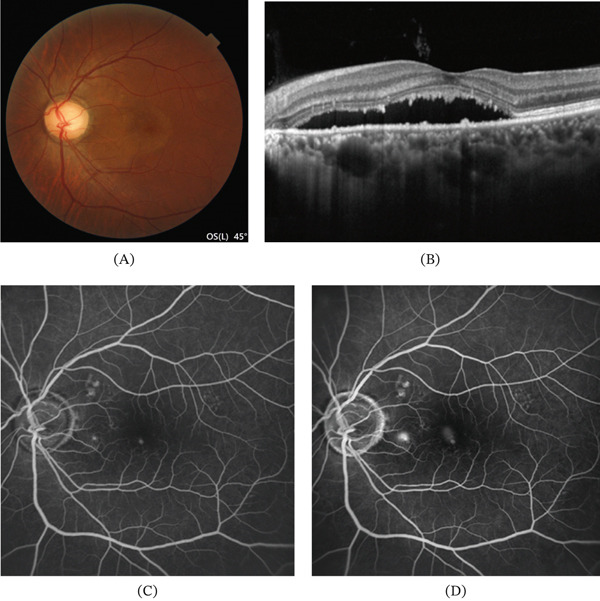
Baseline multimodal imaging of a patient with central serous chorioretinopathy. (A) Color fundus photography and (B) optical coherence tomography revealed serous retinal detachment in the macula. On fluorescein angiography, two areas of focal hyperfluorescence expand and increase in intensity from the (C) early to (D) late phase, indicating leakage.

A man in his 40s, with a past medical history of epididymitis and tonsillitis and taking no current medications, was referred to our tertiary hospital for a 1‐year history of unusual vision in his left eye. On ophthalmologic examination, the best‐corrected visual acuity (BCVA) was 20/25 OS and 20/17 OD (Snellen). Optical coherence tomography revealed macular serous retinal detachment in the left eye (Figure 1A,B). Fluorescein and indocyanine green angiography revealed two focal leakages within the retinal detachment without evidence of uveitis, retinal vasculitis, tumor, or vascular occlusion (Figure 1C,D). After confirming the absence of macular neovascularization using both angiography and optical coherence tomography angiography, the patient was diagnosed with CSC without macular neovascularization. The patient underwent low‐power focal photocoagulation for the two leakage spots to avoid macular atrophy.

One month after the initial visit, the patient presented to the Department of Rheumatology with dysarthria and stomatitis. On suspicion of incomplete Behçet′s disease, the patient was started on prednisone 30 mg daily. A subsequent ophthalmic re‐examination revealed no change in serous retinal detachment, and there were no ocular findings suggestive of Behçet′s disease, such as endophthalmitis or vasculitis. Although the patient′s systemic symptoms improved and the steroid dose was tapered to 8 mg over 5 months, serous retinal detachment persisted. Following fluorescein and indocyanine green angiography (Spectralis, Heidelberg Engineering, Heidelberg, Germany), which confirmed persistent active focal leakage, low‐power focal photocoagulation was performed, which failed to resolve serous retinal detachment. Despite our recommendation of photodynamic therapy, the patient continued to decline treatment because of a lack of visual changes from baseline. Consequently, the serous retinal detachment did not resolve over the following 18 months. Finally, when the BCVA in the left eye worsened to 20/200 and the patient became aware of his mildly decreased vision, photodynamic therapy was administered. As a result, the BCVA improved to 20/100 OS.

**Figure 2:**
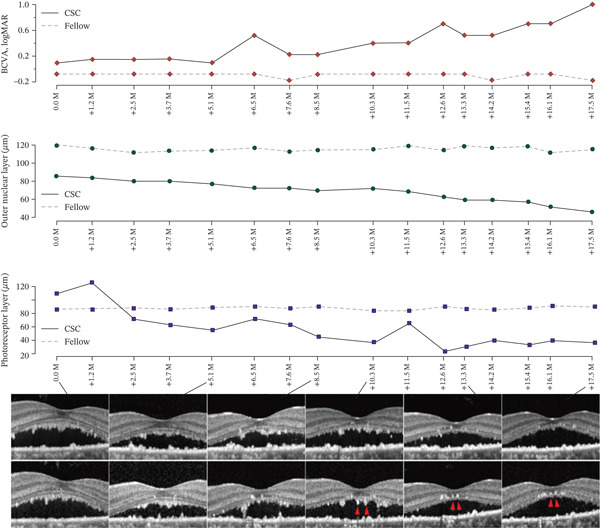
Longitudinal changes in visual acuity and retinal structure. Best‐corrected visual acuity remained stable for the first 5 months and subsequently deteriorated. At baseline, the outer nuclear layer (ONL) in the affected eye was thinner than that in the fellow eye, whereas the photoreceptor layer was thicker. The thicknesses of both the ONL and the photoreceptor layer progressively decreased throughout the follow‐up period. Hyperreflective foci, initially observed beneath the external limiting membrane, appeared to migrate intraretinally over time (arrowhead).

Throughout the 18‐month follow‐up, optical coherence tomography (Spectralis) consistently revealed persistent serous retinal detachment in the affected eye, whereas the fellow eye remained free of exudative changes (Figure 2). The BCVA of the affected eye (OS), initially 20/25, remained stable for the first 5 months before deteriorating to 20/67 at 6 months, 20/100 at 12 months, and 20/200 at 18 months (Snellen).

All thickness measurements were acquired using the automated follow‐up function of spectral domain OCT on a vertical scan passing through the foveal center. At the fovea, we vertically measured the ONL thickness as the distance from the internal limiting membrane to the external limiting membrane (ELM) and the photoreceptor layer thickness as the distance from the ELM to the bottom of the photoreceptor outer segments. At baseline, the subfoveal ONL in the affected eye (OS) was thinner (86 *μ*m) than that in the fellow eye (OD, 120 *μ*m). The ONL in the affected eye progressively thinned, measuring 73 *μ*m at 6 months, 63 *μ*m at 12 months, and 46 *μ*m at 18 months (Figure 2). In contrast, the baseline photoreceptor layer was thicker in the affected eye (OS, 110 *μ*m) than in the fellow eye (OD, 86 *μ*m). The thickness of this layer subsequently decreased to 72 *μ*m at 6 months, 24 *μ*m at 12 months, and 36 *μ*m at 18 months. The optical coherence tomography reflectivity of the photoreceptor layer initially was intermediate but became progressively hyperreflective and developed a focus‐like appearance over time. Eventually, these hyperreflective foci appeared to migrate to the inner side of the sensory retina (Figure 2).

## 3. Discussion

We reported a case of chronic CSC with serous retinal detachment persisting for 18 months. Despite consistently mild visual symptomatic changes throughout, the patient ultimately developed severe vision loss. Baseline measurements in the affected eye revealed reduced ONL thickness and increased photoreceptor layer thickness; subsequently, both layers became progressively thinner. Furthermore, our findings suggest that the intraretinal hyperreflective foci observed in the late stage result from the migration of altered material originating from the photoreceptor layer.

Acute CSC typically presents with mild visual decline that gradually progresses in most cases [2, 7]. In a retrospective study of 217 eyes with chronic CSC, Mrejen et al. [2] reported that there was no significant difference between BCVA at baseline and at the 1‐ and 5‐year follow‐ups; however, the BCVA at 10 years was significantly worse than that at baseline. In our patient, who had a history of steroid use and suspected incomplete Behçet′s disease, BCVA was stable for approximately 5 months before beginning to worsen at a steady rate, a course that indicates relatively early vision loss. Although serous retinal detachment can occur as a rare form of macular involvement in Behçet′s disease, it typically results from retinal vasculitis and is therefore accompanied by cystoid macular edema, a finding that was absent in our patient [8, 9]. Although this case report cannot prove a causal relationship between systemic inflammation or corticosteroid use and rapid BCVA decline, and while steroid use is not an established risk factor for poor visual prognosis, the findings potentially suggest the need for careful clinical monitoring of CSC patients with these underlying conditions [2, 10]. While this case report does not identify the cause of relatively rapid vision loss, it highlights that assuming a slow, multiyear progression for CSC may delay timely intervention in atypical cases.

Previous studies reported that structural damage to the sensory retina was frequently observed in recurrent cases or those with a poor visual prognosis [4, 5]. The ONL contains the cell bodies of photoreceptors, whereas the photoreceptor layer consists of their inner and outer segments. Thinning of these layers and hyperreflectivity of the photoreceptor layer indicate chronicity or severity [4, 5]. However, these associations are based on cross‐sectional, rather than longitudinal, data. In this study, the ONL progressively thinned throughout the follow‐up period. Especially, BCVA began to decline once ONL thickness fell below 80 *μ*m (at 5 months: BCVA 20/25, ONL thickness 78 *μ*m; at 6 months: BCVA 20/67, ONL thickness 73 *μ*m), potentially suggesting that such a threshold might warrant close clinical monitoring during follow‐up. The photoreceptor layer exhibited both thinning and increased reflectivity. Furthermore, we observed that hyperreflective foci originating from the photoreceptor layer migrated into the neurosensory retina. Although structural OCT cannot definitively identify the origin of hyperreflective foci, previously interpreted as lipid/protein deposits, lipid‐laden macrophages, or migratory cells, these findings provide longitudinal evidence of a dynamic pathological process involving photoreceptor cell loss and degeneration [11]. Moreover, the photoreceptor layer was initially thicker than that of the fellow eye before progressing toward thinning, suggesting that photoreceptor outer segment elongation may be an early finding of photoreceptor degeneration.

A patient with CSC and persistent serous retinal detachment may present a relatively rapid decline in BCVA and progressive retinal damage. This chronic condition is associated with specific structural findings, including thinning of the ONL and photoreceptor layer and the development of intraretinal hyperreflective foci.

## Funding

This study was supported by Japan Society for the Promotion of Science (10.13039/501100001691; 24K12746, 24KJ1842).

## Ethics Statement

This study was approved by the Ethics Committee of Kagoshima University (Approval Number: 180243). Due to the retrospective nature of this study, an opt‐out option was provided for study participation. Moreover, during the medical interview, this patient provided informed consent via an opt‐in format for the use of their data for research and case reporting. We omitted nonessential identifying details in this work.

## Conflicts of Interest

The authors declare no conflicts of interest.

## Data Availability

The data that support the findings of this study are available on reasonable requests from the corresponding author. The data are not publicly available due to privacy or ethical restrictions.
